# Supplementary material for: PRMT7 regulates RNA-binding capacity and protein stability in Leishmania parasites
Source: Nucleic Acids Res. 2020 May 4;48(10):5511–26. doi: 10.1093/nar/gkaa211 (PMC7261171; doi:10.1093/nar/gkaa211)

PRMT7 regulates RNA-binding capacity and protein stability in *Leishmania* parasites

Supplemental Information

Supplemental Figure Legends

Supplemental Fig S1. Multiple sequence alignment of all the annotated PRMTs in *L. major* (*Lmj*PRMTs) and *Homo sapiens* (*Hs*PRMTs). The double E loop is part of the catalytic domain of the PRMTs and is important for S-adenosyl-methionine (SAM) and protein substrate binding. The asterisk depicts the *Lmj*PRMT7 E202 residue mutated to generate catalytically inactive E202K and E202Q mutants *in vitro*.

Supplemental Fig S2. Endogenous FLAG-HA-tagging of *L. major* RBPs (**A**) Plasmid map of pFLAG_HA plasmid, synthesized by GenScript for this work, with Alba3 5'flank region (5'FLR) and Alba3^{WGG} mutant sequence in the "RBP-CDS" region. *Sfi*I enzyme sites used (A,B,C,D) produce different overhangs allowing the ligation of four different synthesized DNA sequences at the same time in a specific order. Homologous sequences required for gene replacement are the 5'FLR (500bp) and the protein coding sequence (RBP-CDS, variable). (**B**) Primers that bind to the 5'FLR region of the gene and 3'FLR immediately downstream of the RBP-CDS but absent in the plasmid sequence were used to verify correct replacement of at least one allele by the HA-tagged gene. Expected PCR band sizes are shown for each tagged gene. (**C**) Positive clones were analyzed by PCR amplification of the modified HA-RBP allele (upper bands) and the original unmodified RBP allele (lower bands). DNA marker used was Gene Ruler 1kb (Thermo Fisher).

Supplemental Fig S3. (A) HA-Alba3 protein levels and stability are unaltered in the presence or absence of PRMT7 levels in logarithmic (log)- or stationary (stat)- stage promastigote cells. (**B**)

Cytoplasmic HA-RBP16 levels display a reduced protein half-life in the absence of PRMT7 expression specifically in stat-stage, human-infective promastigotes.

Supplemental Fig S4. **(A)** Western blot examining endogenous levels of *Lmj*RBP16 using anti-*Tb*RBP16 (kind gift of L.Read). *Lmj*RBP16 protein levels are constant in the presence and absence of PRMT7 levels in both log- and stat-phase stages. **(B)** Western blot examining levels of endogenously tagged HA-*Lmj*RBP16 using anti-HA (from Figure 5A). HA-RBP16 protein levels are destabilized specifically in the absence of PRMT7 levels in stationary (stat) cells. **(C)** Weighted Colocalization Coefficients generated from immunofluorescent data **(D-F)** of *Lmj*RBP16 **(D)** or HA-RBP16 **(E)** with Mitotracker mitochondrial marker. *Lmj*RBP16 displays a strong mitochondrial colocalisation (0.65-1.00) while HA-RBP16 shows negligible colocalisation (0.08-0.33; ND = RBP Not Detected). Statistical analyses generated using Zen software (Zeiss, Zinchuk and Zinkchuk, Current Protocols in Cell Biology, 2008). **(D-F)** Immunofluorescence: DAPI (Blue), Mitotracker (Green), RBP (Red), line = 5µm. (**D**) Subcellular localisation of endogenous *Lmj*RBP16 using anti-*Tb*RBP16 shows mitochondrial localisation constant in both lifecycle stages examined. (**E**) Subcellular localisation of endogenously-tagged HA-*Lmj*RBP16 using anti-HA shows cytoplasmic localisation that is destabilized specifically in the absence of PRMT7 levels in stationary (stat) stage promastigote cells. (**F**) Subcellular localisation of endogenously-tagged HA-*Lmj*Alba3 using anti-HA shows cytoplasmic localisation constant in promastigote lifecycle stages.

Supplemental Table Legends

Supplemental Table S1. Global monomethyl arginine peptides identified and quantified by heavy methyl SILAC analysis in WT and Δ*prmt7 Leishmania major*.

Supplemental Table S2. Methylpeptides from RNA-binding proteins (RBPs) that are differentially methylated between WT and Δ*prmt7 Leishmania major*. Proteins were considered RBPs if they present an RNA-binding domain or if they are orthologs of a validated *Trypanosoma brucei* RBP.

Supplemental Table S3. List of oligonucleotides used in this study for PCR or qRT-PCR.

Figure S1

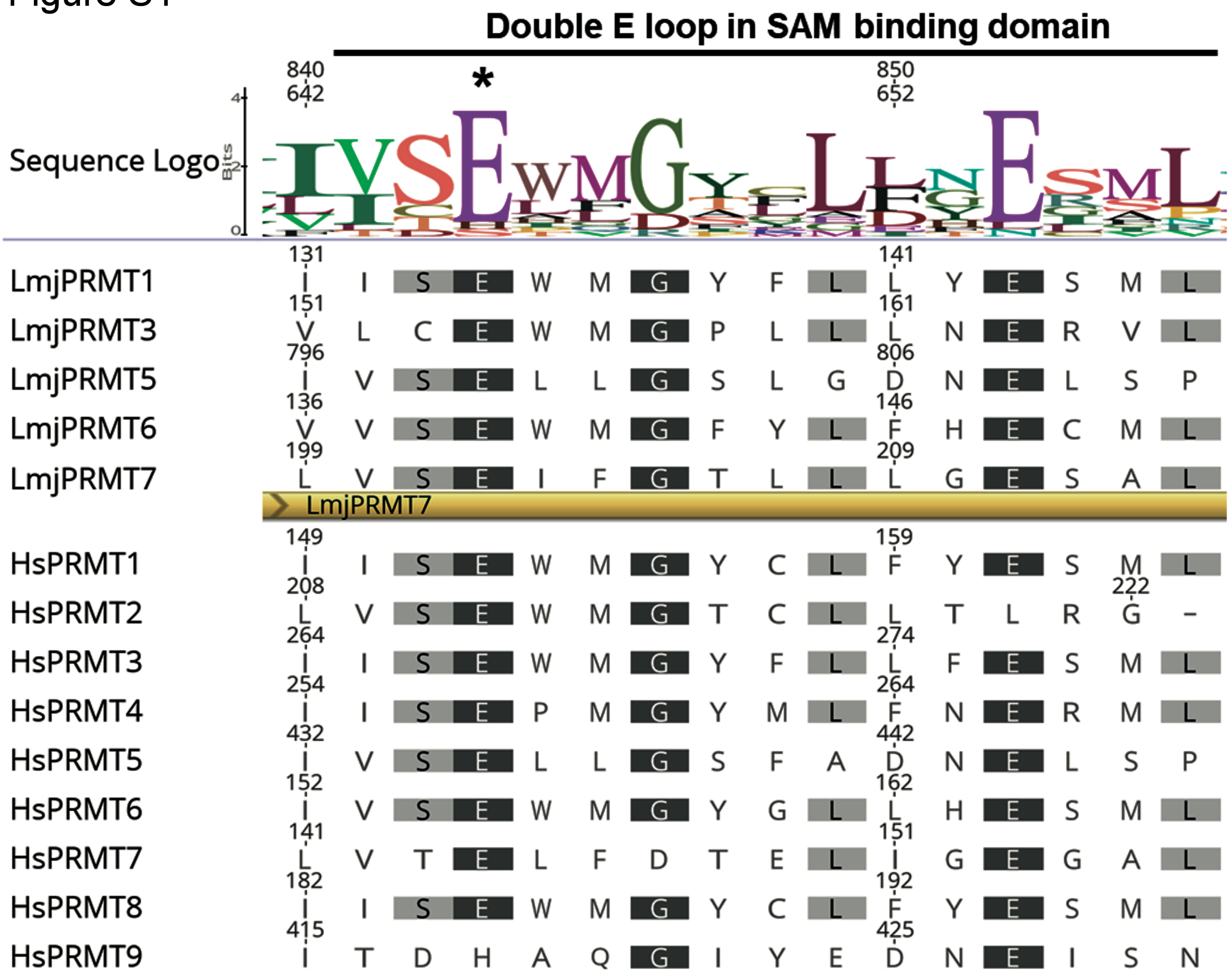

Double E loop in SAM binding domain
Sequence Logo
LmjPRMT1
LmjPRMT3
LmjPRMT5
LmjPRMT6
LmjPRMT7
HsPRMT1
HsPRMT2
HsPRMT3
HsPRMT4
HsPRMT5
HsPRMT6
HsPRMT7
HsPRMT8
HsPRMT9

Figure S2

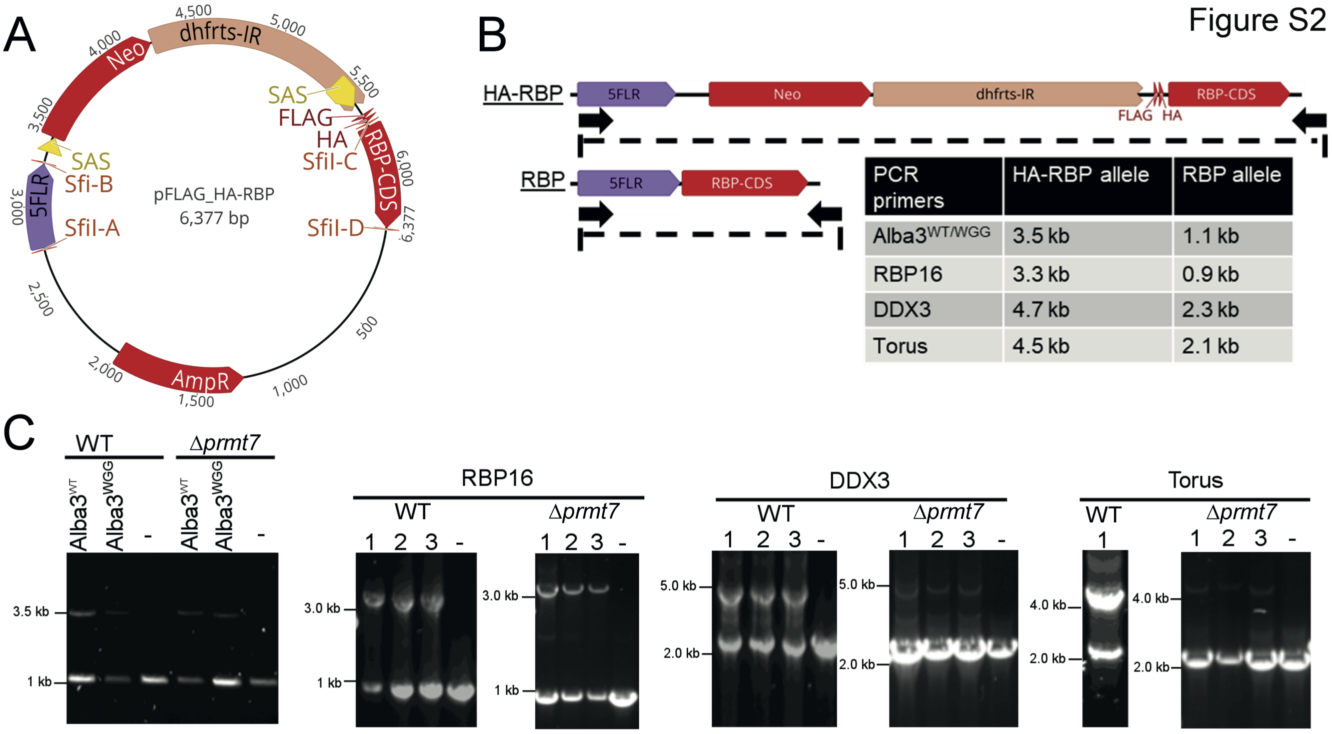

PCR primers	HA-RBP allele	RBP allele
Alba3$^{WT/WGG}$	3.5 kb	1.1 kb
RBP16	3.3 kb	0.9 kb
DDX3	4.7 kb	2.3 kb
Torus	4.5 kb	2.1 kb

Figure S3

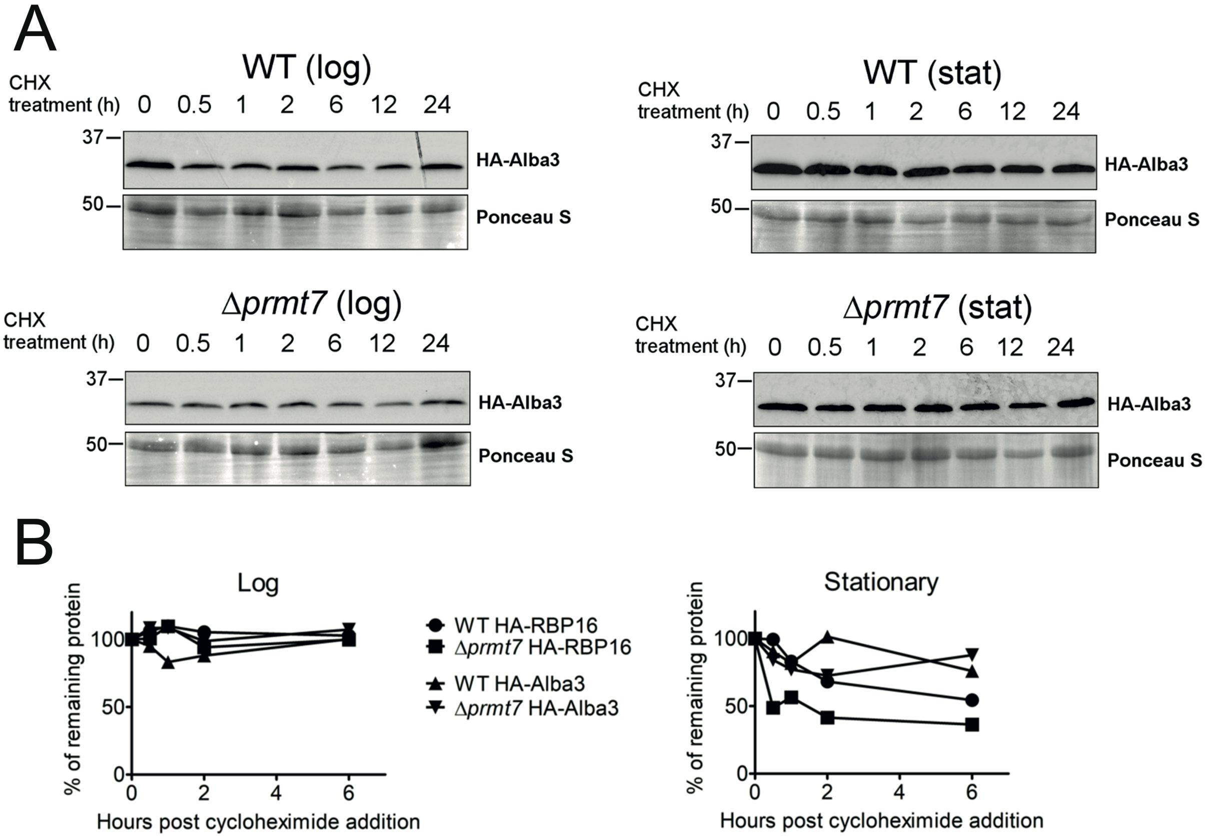
A
WT (log)
CHX treatment (h) 0 0.5 1 2 6 12 24
37
HA-Alba3
50
Ponceau S
WT (stat)
CHX treatment (h) 0 0.5 1 2 6 12 24
37
HA-Alba3
50
Ponceau S
Δprmt7 (log)
CHX treatment (h) 0 0.5 1 2 6 12 24
37
HA-Alba3
50
Ponceau S
Δprmt7 (stat)
CHX treatment (h) 0 0.5 1 2 6 12 24
37
HA-Alba3
50
Ponceau S
B
Log
% of remaining protein
100
50
0
0 2 4 6
Hours post cycloheximide addition
WT HA-RBP16
Δprmt7 HA-RBP16
WT HA-Alba3
Δprmt7 HA-Alba3
Stationary
% of remaining protein
100
50
0
0 2 4 6
Hours post cycloheximide addition

Figure S4

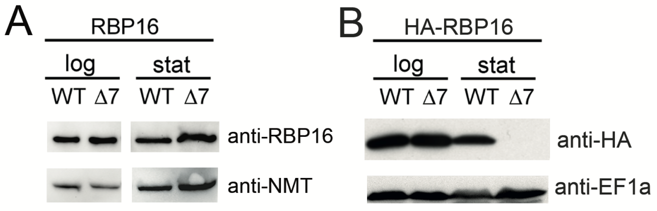

C

Weighted Colocalization with Mitotracker

	log		stat	
	WT	Δ7	WT	Δ7
RBP16	**0.87**	**1.00**	**0.81**	**0.65**
HA-RBP16	**0.33**	**0.29**	**0.10**	**0 (ND)**

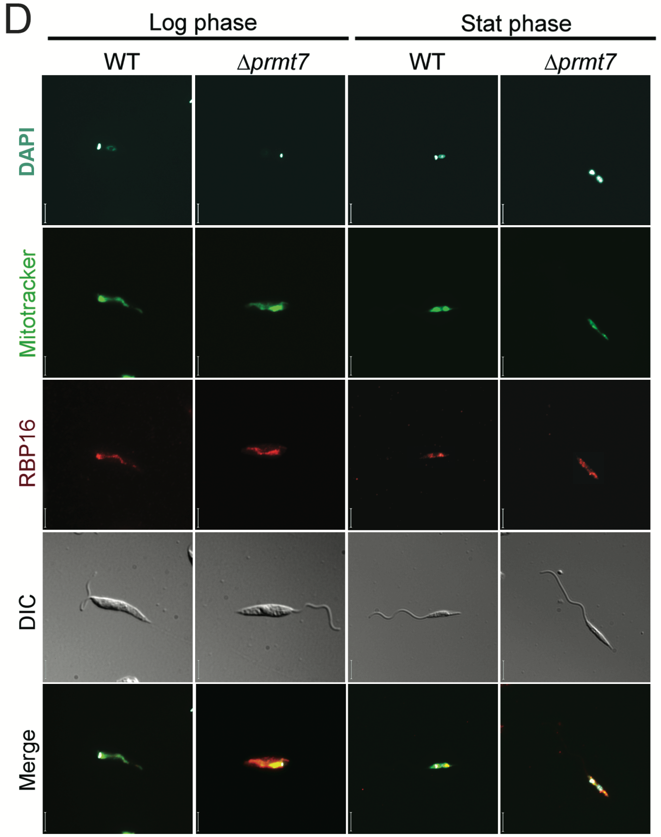

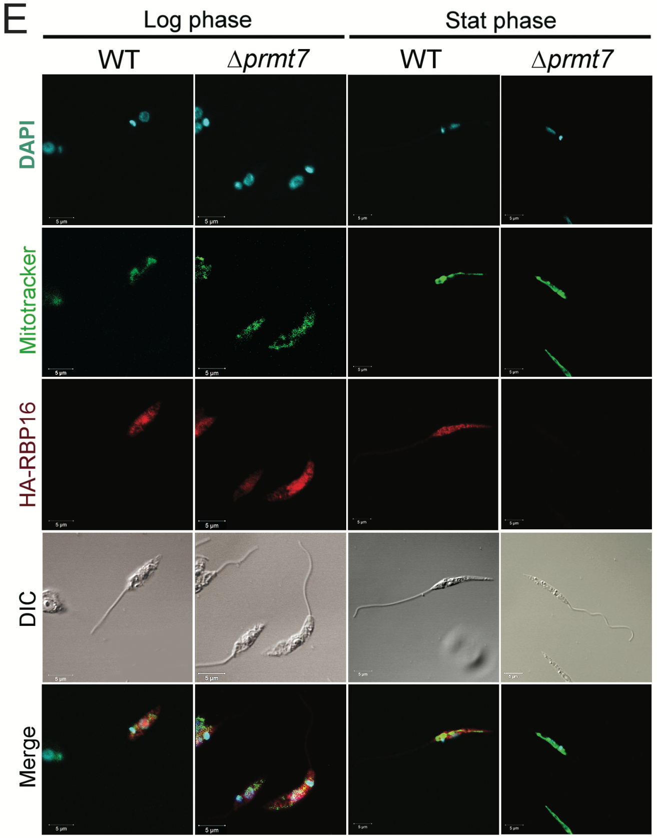

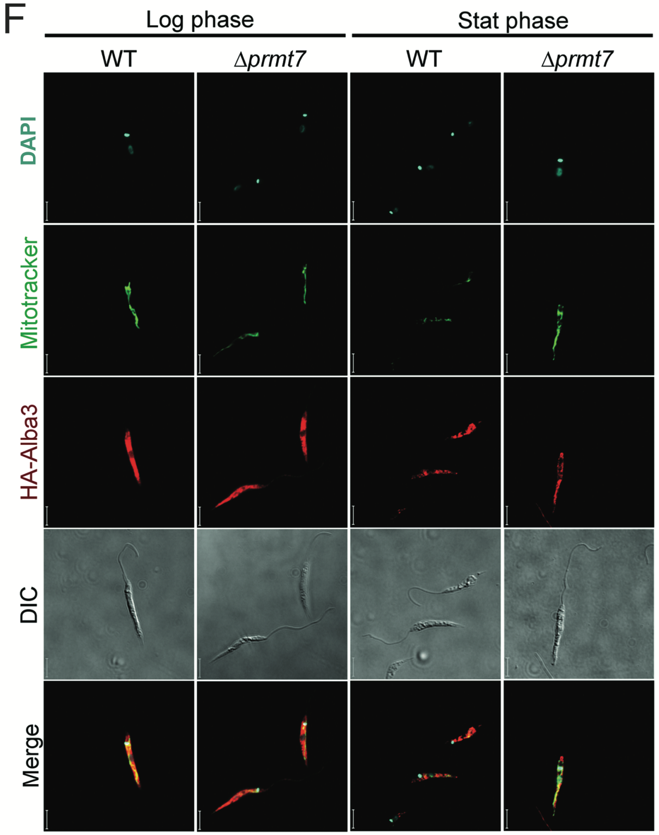

Supplement: gkaa211_Supplemental_Files [file gkaa211_supplemental_files.zip › Ferreira et al NAR_Supplemental_data 0420.pdf]
